# Treatment with an Angiopoietin‐1 mimetic peptide promotes neurological recovery after stroke in diabetic rats

**DOI:** 10.1111/cns.13541

**Published:** 2020-12-21

**Authors:** Poornima Venkat, Ruizhuo Ning, Alex Zacharek, Lauren Culmone, Linlin Liang, Julie Landschoot‐Ward, Michael Chopp

**Affiliations:** ^1^ Department of Neurology Henry Ford Hospital Detroit Michigan USA; ^2^ Department of Physics Oakland University Rochester Michigan USA

**Keywords:** angiopoietin‐1, neuroprotection, neurorestoration, stroke, type 1 diabetes mellitus, vasculotide

## Abstract

**Aim:**

Vasculotide (VT), an angiopoietin‐1 mimetic peptide, exerts neuroprotective effects in type one diabetic (T1DM) rats subjected to ischemic stroke. In this study, we investigated whether delayed VT treatment improves long‐term neurological outcome after stroke in T1DM rats.

**Methods:**

Male Wistar rats were induced with T1DM, subjected to middle cerebral artery occlusion (MCAo) model of stroke, and treated with PBS (control), 2 µg/kg VT, 3 µg/kg VT, or 5.5 µg/kg VT. VT treatment was initiated at 24 h after stroke and administered daily (i.p) for 14 days. We evaluated neurological function, lesion volume, vascular and white matter remodeling, and inflammation in the ischemic brain. In vitro, we evaluated the effects of VT on endothelial cell capillary tube formation and inflammatory responses of primary cortical neurons (PCN) and macrophages.

**Results:**

Treatment of T1DM‐stroke with 3 µg/kg VT but not 2 µg/kg or 5.5 µg/kg significantly improves neurological function and decreases infarct volume and cell death compared to control T1DM‐stroke rats. Thus, 3 µg/kg VT dose was employed in all subsequent in vivo analysis. VT treatment significantly increases axon and myelin density, decreases demyelination, decreases white matter injury, increases number of oligodendrocytes, and increases vascular density in the ischemic border zone of T1DM stroke rats. VT treatment significantly decreases MMP9 expression and decreases the number of M1 macrophages in the ischemic brain of T1DM‐stroke rats. In vitro, VT treatment significantly decreases endothelial cell death and decreases MCP‐1, endothelin‐1, and VEGF expression under high glucose (HG) and ischemic conditions and significantly increases capillary tube formation under HG conditions when compared to non‐treated control group. VT treatment significantly decreases inflammatory factor expression such as MMP9 and MCP‐1 in macrophages subjected to LPS activation and significantly decreases IL‐1β and MMP9 expression in PCN subjected to ischemia under HG conditions.

**Conclusion:**

Delayed VT treatment (24 h after stroke) significantly improves neurological function, promotes vascular and white matter remodeling, and decreases inflammation in the ischemic brain after stroke in T1DM rats.

## Graphical abstract

Vasculotide treatment initiated at 24 h after stroke significantly improves neurological function, promotes vascular and white matter remodeling and decreases inflammation in the ischemic brain after stroke in T1DM rats.

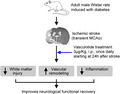

## 1 INTRODUCTION

Ischemic stroke remains a leading cause of death and long‐term disability worldwide.^1^ Diabetes mellitus (DM) increases ischemic stroke incidence at all ages and is an independent risk factor for stroke recurrence.^1^ Patients with DM at stroke onset have higher risk of death and exhibit aggravated stroke pathology mostly derived from extensive metabolic abnormalities, vascular damage, white matter deterioration, and an inflammatory milieu that, in concert, hinders recovery.^1^, ^2^, ^3^ Thus, there is a need for clinical and research effort to mitigate neurological disability and improve functional recovery after stroke especially in patients with comorbidities like DM.

Vasculotide (VT) is an angiopoietin‐1 (Ang1) mimetic peptide. Ang1 is an endothelial growth factor that is essential for endothelial cell survival and function, endothelial cell migration and adhesion, and vessel maturation during angiogenesis.^4^, ^5^ In ischemic stroke patients, low circulating Ang1 levels are associated with poor stroke outcome, that is, greater disability and higher mortality at 3 months after stroke.^6^ Pre‐clinical studies have reported that therapeutic interventions that increase Ang1 are associated with smaller infarcts, improved blood‐brain barrier (BBB) integrity, and recovery of neurological function.^7^, ^8^ Ang1 is also known to exert anti‐inflammatory effects and to protect endothelial cell permeability against inflammatory factors.^4^ Comorbidity of diabetes and stroke impairs Ang1‐mediated vascular remodeling.^9^, ^10^ In T1DM mice, hyperglycemia affects cerebral microcirculation, increases cortical microvessel density, induces smooth muscle and endothelial cell dysfunction, decreases tight junction proteins, and decreases the expression of angiogenic factor such as Ang1, vascular endothelial growth factor (VEGF), transforming growth factor‐β, and platelet‐derived growth factor‐β compared to non‐DM mice.^11^ When subject to stroke, T1DM mice exhibit greater lesion volume, severe neurological deficits, increased BBB permeability, and delayed angiogenesis compared to non‐DM mice.^11^ In diabetic stroke mice, decreased Ang1 in ischemic brain has been associated with poor functional outcome, increased BBB disruption, and brain hemorrhagic transformation.^10^ Treatment with VT has been shown to improve endothelial cell survival and migration and accelerate diabetic wound healing in mice.^12^ In our previous study, we found that VT pre‐treatment of stroke in rats with type 1 diabetes mellitus (T1DM) exerts neuroprotective effects by decreasing the expression of pro‐inflammatory factors in the ischemic brain.^13^ While the neuroprotective effects of Ang1 treatment in non‐diabetic stroke are well documented,^7^, ^8^, ^14^, ^15^ there are few studies investigating the therapeutic effects of Ang‐1 in diabetic stroke.^13^, ^14^ The therapeutic efficacy and long‐term effects of delayed treatment with VT in T1DM rats subjected to stroke remain to be determined. In this study, we employ a rodent model of stroke in T1DM rats and identify the therapeutic dose and long‐term effects of delayed VT treatment on neurological function, inflammatory responses, and vascular and white matter remodeling.

## 2 MATERIAL AND METHODS

All experimental procedures were carried out in accordance with the National Institutes of Health (NIH) Guide for the Care and Use of Laboratory Animals and were approved by the Institutional Animal Care and Use Committee (IACUC) of the Henry Ford Health System. This manuscript is prepared following ARRIVE guidelines.^16^

### 2.1 Animal model and experimental groups

To induce T1DM in adult male Wistar rats (225–250 g, 8–12 weeks old; Charles River), a single dose of Streptozotocin (STZ, i.p., 60 mg/kg, Enzo) was employed. Since STZ dose dependently damages pancreatic β cells, rats develop T1DM and have high glucose (HG) levels within approximately 2 weeks after STZ injection.^17^ Rats with fasting blood glucose >300 mg/dl (AgaMatrix Advanced blood glucose monitoring system) were included in the study and were subjected to a transient (2 h) middle cerebral artery occlusion (MCAo) stroke model, as previously described.^18^ T1DM rats were randomized at 1 day after stroke and assigned to one of four groups: PBS‐treated control (*n* = 7); 2 µg/kg VT (*n* = 7); 3 µg/kg VT (*n* = 8); or 5.5 µg/kg VT (*n* = 6). To verify the therapeutic effects of 3 µg/kg VT in non‐DM stroke rats, adult male Wistar rats (225–250 g, 8–12 weeks old; Charles River) were subjected to 2 h MCAo and randomized to Control (*n* = 5) or 3 µg/kg VT (*n* = 6) treatment group. All rats were sacrificed at 14 days after MCAo for immunostaining quantification analysis.

Previous studies have reported that a dose of 10 µg/kg (200 ng/mouse) VT reduces acute skin radiation damage,^19^ a dose of 200 ng VT significantly reduced mortality in a murine sepsis model,^20^ 500 ng VT/mice exerts protection against a murine model of severe influenza,^21^ and administering 3 µg/kg VT half an hour prior to MCAo, and at 8 and 24 h after MCAo induces neuroprotection in T1DM stroke rats.^13^ Employing high doses of VT can result in a suboptimal activation of Tie2 receptor and decreased biological efficacy in vivo,^12^ while lower concentrations of Ang1 improve Tie2 activation relative to high concentrations.^22^, ^23^ Therefore, based on body surface area (BSA) calculations,^24^ we tested three doses (5.5, 3, and 2 µg/kg) in this study. Route of administration and dosing interval was chosen based on previous studies that have indicated that i.p administration of VT (but not oral administration) in healthy mice significantly increases endothelial Tie2 activation up to 72 h after injection and increases plasma levels of VT at 24 h after injection which declined to basal levels by 96 h after injection.^20^, ^21^ Therefore, VT treatment was initiated at 24 h after stroke and administered once daily via i.p. injection.

### 2.2 Function testing

To evaluate neurological functional outcome after stroke and the therapeutic efficacy of VT treatment, all rats were subjected to modified neurological severity score (mNSS) testing on days 1, 7, and 14 after MCAo, conducted by an investigator blinded to the experimental groups. The mNSS test is widely used to evaluate neurological function following brain injury in rodents and is a composite of motor, sensory, balance, and reflex tests.^25^ The absence of a tested reflex or abnormal response is scored as one point. Neurological function is scored between 0–18 with 0 indicating no deficits and 18 indicating maximum deficits. Rats with mNSS score <6, indicating a small to no lesion, were excluded from the study because their condition improves regardless of treatment. Rats with mNSS equal to or greater than 13 have poor survival, and their condition deteriorates regardless of treatment and also were not included in the study.

### 2.3 Lesion volume and immunohistochemistry

All brains were fixed by transcardial perfusion with 0.9% saline, followed by perfusion and immersion in 4% paraformaldehyde, and were then embedded in paraffin. Seven coronal brain sections were processed from each brain, and a series of 6 μm thick sections were cut from blocks which were obtained from the center of the lesion (bregma −1 mm to +1 mm). Hematoxylin and eosin (H&E) staining was used for ischemic lesion volume calculation, and lesion volume is presented as a percentage of the lesion compared with the contralateral hemisphere. Bielschowsky silver (BS) staining was used to stain axons, and Luxol fast blue (LFB) was used to stain myelin. Antibodies against MMP9 (Matrix metalloproteinase 9, 1:500; Santa Cruz Biotechnology), ED1 (microglia/macrophages marker, 1:30; AbDSerotec), TUNEL (terminal deoxynucleotidyl transferase dUTP nick end labeling; Millipore Sigma), MBP (myelin basic protein, 1:250; Millipore Sigma), SMI‐32 (Sternberger monoclonal clone 32, 1:1000; BioLegend), NG2 (neuron‐glial antigen 2, 1:400; Millipore Sigma), and vWF (von Willebrand factor, an endothelial cell marker, 1:400; Dako) were employed. Control experiments consisted of staining brain coronal tissue sections as outlined above, but without addition of primary antibody.

### 2.4 Quantification analysis

All immunostaining quantification analysis was performed by an investigator blinded to the experimental groups. Five slides from each brain (each slide containing eight fields of the ischemic boundary zone [IBZ]) were digitized under a 20× objective (Olympus BX40) using a 3‐CCD color video camera (Sony DXC‐970MD) interfaced with an MCID image analysis system (Imaging Research). For each field of view, the numbers of positive cells were counted or the positive‐stained areas were measured using a built‐in densitometry function (MCID image analysis system) with density threshold above unstained set uniformly for all groups.

### 2.5 Endothelial cell culture and oxygen glucose deprivation

Mouse brain endothelial cells (ATCC, catalog #CRL‐2299) were cultured, and cells were subjected to oxygen glucose deprivation (OGD). Serum and glucose free media were used, and cells were placed in a hypoxia chamber (Forma Anaerobic System; Thermo Scientific) with 37°C incubator for 2 h. After 2 h, the cells were removed from the chamber and media was replaced with HG (37.5 mM) culture media.

### 2.6 Lactate dehydrogenase cytotoxicity assay

Five hundred cells in 100 µl of media were plated in a 96‐well plate and incubated for 48 h. Media from secreted LDH wells was collected, and two freeze/thaw cycles were implemented before collecting media which was then diluted with water and added to a fresh plate. LDH assay was performed using the CytoTox 96 Non‐Radioactive Cytotoxicity Assay kit (Promega) following standard protocol. The absorbance was recorded at 490 nm (Molecular Devices). Previous studies have investigated the dose‐dependent phosphorylation of the Tie2 receptor by VT (1, 10, 100, and 1000 ng/ml) in primary lung microvascular endothelial cells as well as kidney glomerular endothelial cells, and a dose of 10 ng/ml VT significantly phosphorylated the Tie2 receptor in endothelial cells.^26^, ^27^ Therefore, 10 ng/ml is employed. The experimental groups included: (1) HG, (2) HG + OGD, and (3) HG + OGD + 10 ng/ml VT.

### 2.7 Angiogenesis protein array

Protein was extracted using TRIzol Reagent (Thermo Fisher Scientific). Angiogenesis antibody array analysis was performed using the Mouse Angiogenesis Array kit (R&D Systems, ARY015; detecting 53 proteins) according to manufacturer's protocol. Briefly, 200 µg protein/membrane was hybridized on the antibody array membrane at 4°C overnight. A 1:5000 dilution of Streptavidin‐HRP was used for detection. Membranes were imaged using the Protein Simple FluorChem E system, and data were analyzed using ImageJ and normalized using internal controls. The experimental groups included: (1) HG, (2) HG + OGD, and (3) HG + OGD + 10 ng/ml VT.

### 2.8 Endothelial cell capillary tube formation assay

Matrigel (Becton Dickinson) was diluted to 75% with SF DMEM, and 100 µl was added per well in a 96‐well plate before being incubated at 37˚C for 30 min. A total of 22,500 cells/100 µl with or without VT treatment were added to each well (*n* = 4 wells/group) and allowed to incubate for 3 h after which the Matrigel wells were digitized under a 10 × objective (Olympus BX40). To measure total tube length of capillary tube formation, tracks of endothelial cells organized into networks of cellular cords (tubes) were counted and averaged in four randomly selected microscopic fields. The experimental groups included: (1) HG and (2) HG + 10 nM VT.

### 2.9 Primary cortical neuron (PCN) culture

Primary cortical neurons were harvested from pregnant (day 18) embryonic Wistar rats (Charles River). The cultures were prepared as previously described with some modifications.^28^ Briefly, the embryo cerebral cortex was dissected and dissociated in Ca^2+^ and Mg^2+^ free HBSS with 0.125% trypsin. The cells were placed on poly‐d‐lysine (Sigma) coated dishes (35 mm; Corning) and initially cultured in DMEM media (Life Technologies) with 5% fetal bovine serum for 5 h, then neurobasal growth medium (Life Technologies) with 2% B‐27 (Life Technologies), 2 mM GlutaMax, and 1% antibiotic‐antimycotic were used. To subject PCN's to OGD, serum and glucose free media were used, and cells were placed in a hypoxia chamber (Forma Anaerobic System; Thermo Scientific) with a 37°C incubator for 2 h. After 2 h, the cells were removed from the chamber and media was replaced with HG (37.5 mM) culture media. The experimental groups included: (1) HG + OGD and (2) HG + OGD+10 ng/ml VT.

### 2.10 Macrophage culture

Macrophages were derived from bone marrow cells, as previously described.^29^ The tibias of three male, young (3–4 months old) C57BL/6 mice were flushed with a syringe fitted with a 20G needle in order to release bone marrow aspirates. Isolated bone marrow cells were suspended in DMEM with 10% FBS and 1 mg/ml each of penicillin and streptomycin. The cells were gently lifted, centrifuged, and plated onto a type I collagen‐coated 10 cm dish in RPMI media (StemXVivo; R&D Systems) supplemented with 100 µg/ml each of penicillin and streptomycin. The cells were differentiated in a humidified incubator with 5% CO_2_ at 37°C, and macrophages were fully differentiated after 6 days. At day 7, the media was collected, and cells were harvested with 0.25% trypsin. Cells were then centrifuged, counted, and resuspended in media. The cells were then replated on a 6‐well plated with half old and half fresh media and cultured for 1 day. Cells were grown to 80%–90% confluency, treated with or without LPS (100 ng/ml), and incubated at 37°C in a 5% CO_2_ chamber for 24 h. The experimental groups included: (1) Control, (2) +10 nM VT, (3) LPS, and (4) LPS + 10 nM VT.

### 2.11 Real‐time polymerase chain reaction

RNA extraction was performed using TRIzol reagent (Invitrogen) according to standard protocol. cDNA was made with 2 µg of total RNA using the M‐MLV (Invitrogen) protocol, and 2 µl of cDNA was subsequently used to run a quantitative PCR using the SYBR Green RT‐PCR method. PCR was performed on a ViiA 7 PCR instrument (Applied Biosystems) using 3‐stage program parameters provided by the manufacturer as follows: 2 min at 50°C, 10 min at 95°C, and then 40 cycles of 15 s at 95°C and 1 min at 60°C. Each sample was tested in triplicate, and analysis of relative gene expression was performed using the $2^{‐\Delta \Delta C_{t}}$ method.^30^ The following primers were used:

IL‐1β: Fwd: TGTCTGACCCATGTGAGCTG; Rev: CCCAAGGCCACAGGGATTTT

MMP9: Fwd: ACACGGATCCCCCAACCTTT; Rev: AGGTCAGAACCGACCCTACAA

MCP‐1: Fwd: TGTCACCAAGCTCAAGAGAGAG; Rev: CTGAAGTCCTTAGGGTTGATGC

### 2.12 Statistical analysis

Statistical analysis was performed using the GraphPad Prism 8 software. Gaussian distribution of the values in each experimental group was assessed using Shapiro‐Wilk (for *n* < 8) or D’Agostino‐Pearson (for *n* > 8) normality tests. For immunostaining analysis, all comparisons between two groups with a normal distribution were performed using unpaired Student's *t*‐test with Welch's correction, and for groups without normal distribution, nonparametric, two‐tailed Mann‐Whitney test was used. For mNSS test, two‐way analysis of variance (ANOVA) accompanied by Tukey's post hoc test was used. A *p*‐value < 0.05 was considered statistically significant. All data are expressed as mean ± SE.

## 3 RESULTS

### 3.1 In T1DM rats subjected to stroke, VT treatment dose dependently improves neurological functional outcome

**Figure 1:**
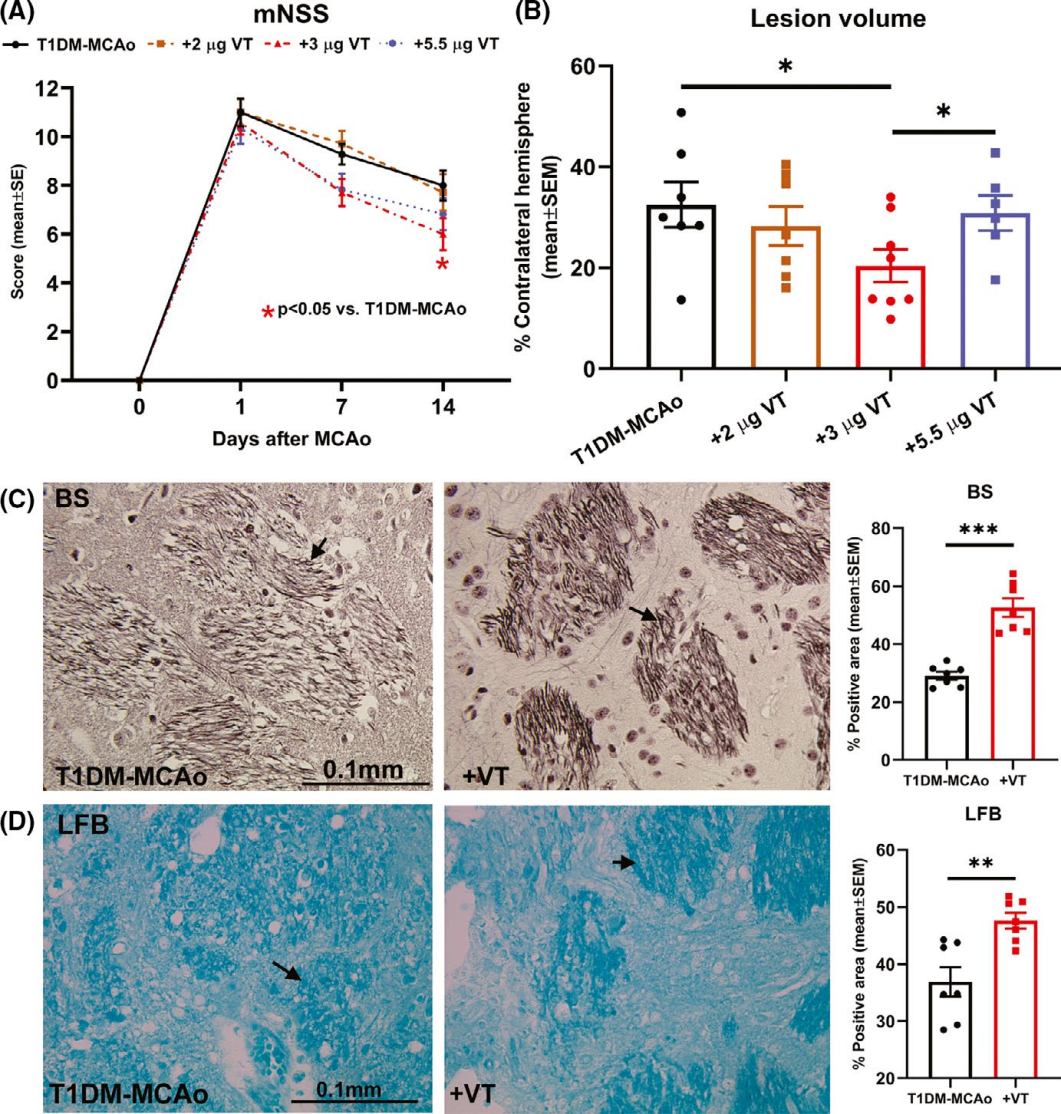
(A) Neurological function was evaluated on days 1, 7, and 14 after stroke using modified neurological severity score (mNSS) test and analyzed using 2‐way ANOVA and Tukey's post hoc test. Treatment of T1DM stroke with 3 µg/kg VT initiated 24 h after stroke significantly improves neurological functional outcome compared to PBS‐treated T1DM‐MCAo rats. (B) At 14 days after stroke, lesion volume was evaluated using H&E staining and analyzed using unpaired Student's *t*‐test with Welch's correction. Treatment of T1DM stroke with 3 µg/kg VT initiated 24 h after stroke significantly decreases lesion volume compared to PBS‐treated or 5.5 µg/kg VT‐treated T1DM‐MCAo rats. Therefore, 3 µg/kg VT is identified as a therapeutic dose and used for subsequent analysis. (C‐D) Bielschowsky silver (BS) and Luxol fast blue (LFB) staining were employed to evaluate axon and myelin density and analyzed using unpaired Student's *t*‐test with Welch's correction. Treatment of T1DM stroke with 3 µg/kg VT significantly increases axon density and myelin density in the striatal white matter in ischemic boundary zone compared to PBS‐treated T1DM‐MCAo rats. Arrows indicate positive staining. *N* = 6–8 animals per group. *p < 0.05, **p < 0.01, ***p < 0.001

To identify the therapeutic dose of VT in T1DM stroke rats, we employed three different doses 2, 3, and 5.5 µg/kg and treatments were initiated at 24 h after stroke and administered once daily via i.p. injection. Neurological function was evaluated at 1, 7, and 14 days after MCAo using mNSS test. Rats were sacrificed at 14 days after stroke, and we evaluated whether VT treatment reduces infarct volume using H&E staining. Figure 1A shows that 3 µg/kg VT treatment significantly (*p* < 0.05) improves neurological function compared to PBS‐treated T1DM stroke rats. Figure 1B shows that 3 µg/kg VT treatment significantly (*p* < 0.05) decreases ischemic lesion volume compared to 2 µg/kg VT, 5.5 µg/kg VT, or PBS‐treated T1DM stroke rats. However, 2 µg/kg VT or 5.5 µg/kg VT treatments did not significantly improve neurological function or reduce lesion volume compared to control T1DM stroke rats. Therefore, 3 µg/kg VT is identified as a therapeutic dose and used for subsequent analysis.

To verify the therapeutic effects of VT in non‐DM stroke rats, male Wistar rats were subjected to MCAo and treated with 3 µg/kg VT. Neurological function was evaluated on days 1, 7, and 14 after stroke using mNSS test. Figure S1A shows that treatment of non‐DM stroke rats with 3 µg/kg VT initiated 24 h after stroke and administered once daily significantly (*p* < 0.01 on day 7, *p* = 0.08 on day 14) improves neurological functional outcome compared to control MCAo rats. At 14 days after stroke, lesion volume was evaluated using H&E staining. Figure S1B shows that treatment of stroke with 3 µg/kg VT initiated 24 h after stroke significantly (*p* < 0.01) decreases lesion volume compared to control MCAo rats.

### 3.2 In T1DM stroke rats, 3 µg/kg VT treatment significantly promotes white matter integrity in the ischemic brain

**Figure 2:**
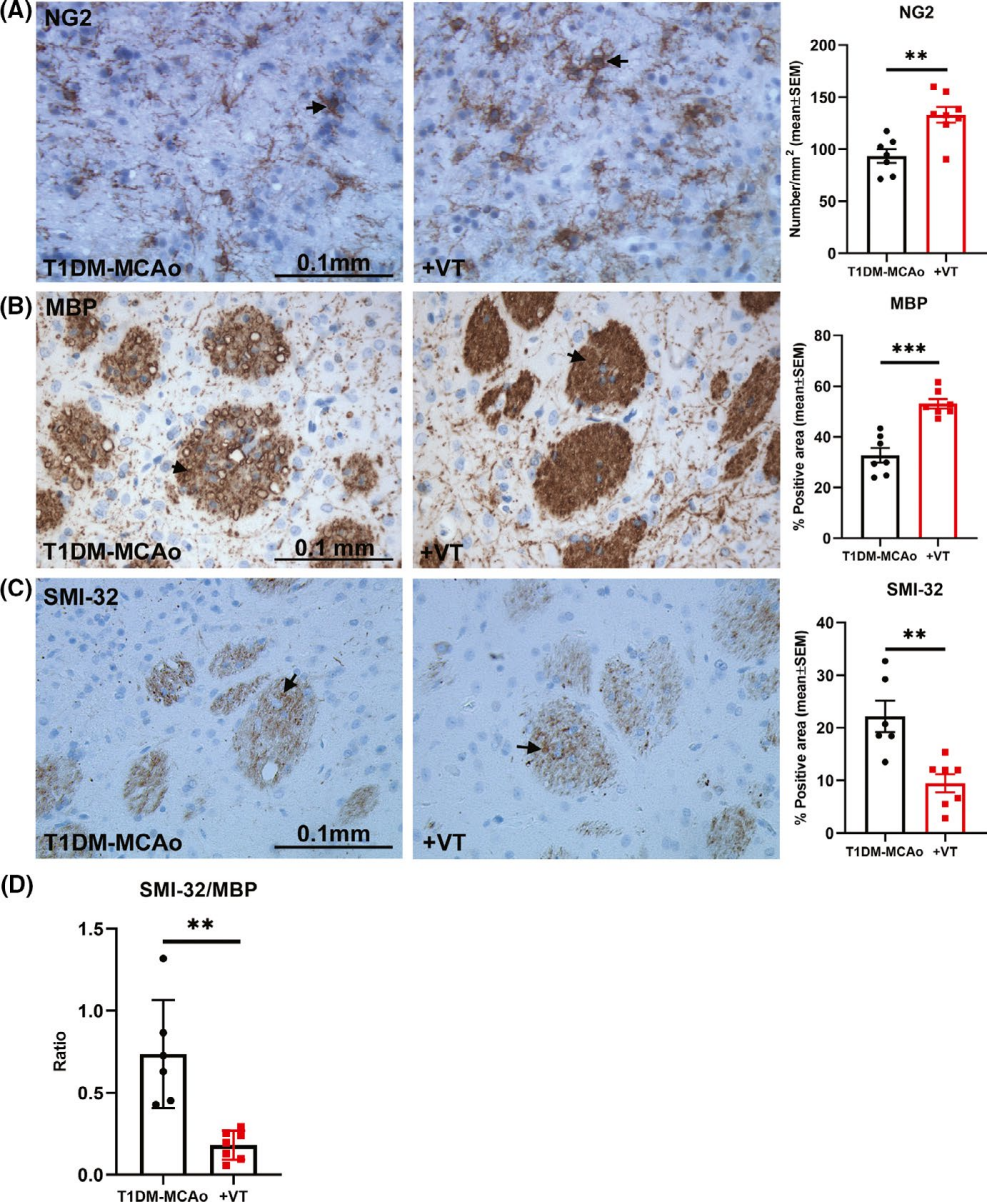
(A) NG2 immunostaining was analyzed using unpaired Student's *t*‐test with Welch's correction to measure the number of oligodendrocyte progenitor cells in the ischemic border zone. Treatment of T1DM stroke with 3 µg/kg VT significantly increases the number of NG2 positive oligodendrocyte progenitor cells compared to PBS‐treated T1DM‐MCAo rats. (B‐D) Immunostaining with Myelin basic protein (MBP) and Sternberger monoclonal clone 32 (SMI‐32) was used to evaluate the integrity of myelin and degree of demyelination, respective and analyzed using unpaired Student's *t*‐test with Welch's correction. Treatment of T1DM stroke with 3 µg/kg VT significantly increases MBP expression and decreases SMI‐32 expression in the striatal white matter in ischemic boundary zone compared to PBS‐treated T1DM‐MCAo rats. The ratio of the SMI‐32/MBP was significantly decreased in T1DM stroke rats receiving 3 µg/kg VT treatment compared to PBS treatment, indicating that VT treatment improves white matter integrity in T1DM stroke rats. Arrows indicate positive staining. *N* = 6–8 animals per group. **p < 0.01, ***p < 0.001

White matter injury and impaired oligodendrogenesis in diabetic stroke are known to contribute to poor long‐term functional recovery.^31^ To test the effects of delayed VT treatment on white matter integrity after stroke in T1DM rats, we employed BS, LFB, and MBP staining as well as SMI‐32 and NG2 immunostaining. Our data indicate that 3 µg/kg VT treatment in T1DM stroke rats significantly increases axon density (BS, *p* < 0.001, Figure 1C), myelin density (LFB, *p* < 0.01, Figure 1D), and number of oligodendrocyte progenitor cells (NG2, *p* < 0.01, Figure 2A) in the IBZ compared to PBS‐treated T1DM stroke rats. MBP is a marker for the integrity of myelin sheath, SMI‐32 is a marker for demyelinated axons, and an increase in the ratio of SMI‐32 to MBP is an established indicator of white matter injury.^32^, ^33^, ^34^, ^35^, ^36^ Our data also indicate that 3 µg/kg VT treatment in T1DM stroke rats significantly increases MBP(*p* < 0.001, Figure 2B), decreases SMI‐32 (*p* < 0.01, Figure 2C), and decreases SMI‐32/MBP ratio (*p* < 0.01, Figure 2D) in the IBZ compared to PBS‐treated T1DM stroke rats. Therefore, VT treatment significantly improves white matter injury after stroke in T1DM rats.

### 3.3 VT significantly increases vascular density in the ischemic brain of T1DM stroke rats as well as promotes capillary tube formation in vitro

**Figure 3:**
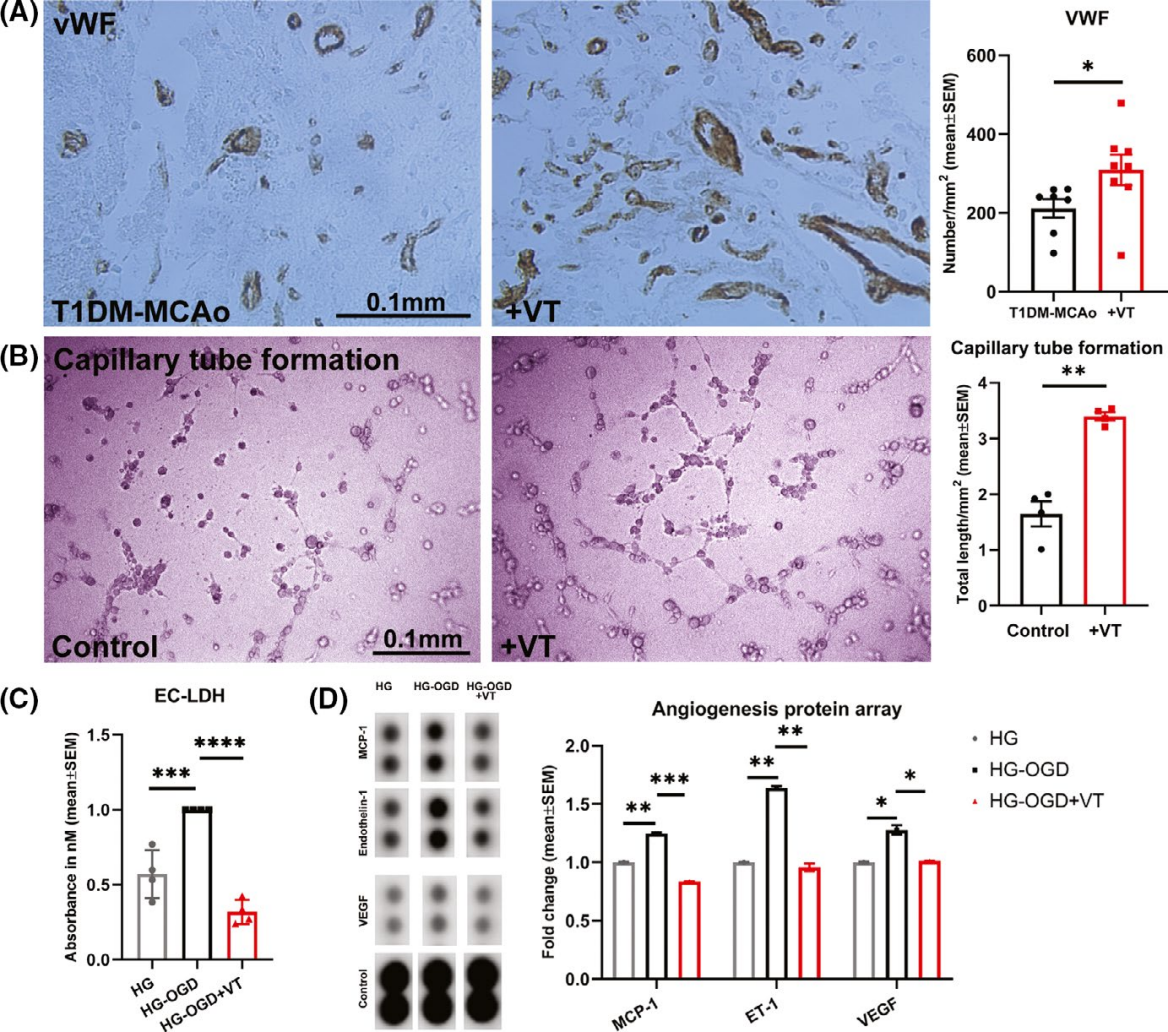
(A) Immunohistochemical staining for von Willebrand factor (vWF) was used to evaluate vascular density and data analyzed with non parametric, two‐tailed Mann‐Whitney test. Treatment of T1DM stroke with 3 µg/kg VT initiated 24 h after stroke significantly increases vascular density in the ischemic boundary zone compared to PBS‐treated T1DM‐MCAo rats. *N* = 7–8 animals per group. *p < 0.05. (B) To confirm angiogenic effects of VT treatment in vitro, endothelial cell capillary tube formation assay was employed and analyzed using unpaired Student's *t*‐test with Welch's correction. Under high glucose (HG) conditions, 10 nM VT treatment significantly increases total length of capillary tube formation, that is, tracks of endothelial cells organized into networks of cellular cords or tubes compared to untreated control group. *n* = 4 wells/group, **p < 0.01. (C) To evaluate the effect of VT treatment on endothelial cell toxicity, LDH assay followed by one‐way ANOVA and Tukey's post hoc test was performed. Treatment with 10 ng/ml VT significantly decreases endothelial cell toxicity under conditions of HG and oxygen glucose deprivation compared to non‐treated control group. *n* = 4 wells/group, ***p < 0.001, ****p < 0.0001. (D) To evaluate the differential expression of angiogenic factors by endothelial cells following VT treatment, we employed an angiogenesis protein array and data were analyzed using ImageJ and normalized using internal controls. Under HG conditions, oxygen glucose deprivation significantly increases endothelial cell expression of MCP‐1, Endothelin‐1 (ET‐1) and vascular endothelial growth factor (VEGF) compared to HG alone group. Treatment of endothelial cells subjected to HG and oxygen glucose deprivation with 10 ng/ml VT significantly decreases MCP‐1, ET‐1, and VEGF levels compared to non‐treated control group. *p < 0.05, **p < 0.01, ***p < 0.001

In diabetic stroke, severe reduction in cerebral blood flow, impaired recruitment of collateral vessels, abnormal vascular sprouting, and dysfunctional angiogenesis are known to contribute to poor stroke outcome.^3^, ^37^ To evaluate whether VT treatment regulates post‐stroke angiogenesis, vascular density was measured using vWF immunostaining in the IBZ of T1DM‐stroke rats, and an in vitro capillary tube formation assay was employed under HG conditions. Our data indicate that 3 µg/kg VT treatment in T1DM stroke rats significantly increases the number of vessels (vWF, *p* < 0.05, Figure 3A) in the IBZ compared to PBS‐treated T1DM stroke rats. Figure 3B shows that 10 nM VT treatment significantly improves endothelial cell capillary tube formation compared to non‐treated control group. To test whether VT treatment rescues endothelial cell death under conditions of HG and ischemia, we performed LDH assay. Figure 3C shows that under HG conditions, OGD significantly increases endothelial cell death (*p* < 0.001) compared to non‐ischemic group, while 10 ng/ml VT treatment significantly (*p* < 0.0001) decreases OGD‐induced endothelial cell toxicity compared to non‐treated OGD group. We then employed an angiogenesis protein array to compare the expression of various angiogenic factors. Figure 3D shows that in endothelial cells subject to HG and OGD conditions, monocyte chemoattractant protein‐1 (MCP‐1), endothelin‐1 (ET‐1), and VEGF expression were significantly (*p* < 0.05) increased compared to HG control. Treatment with 10 ng/ml VT significantly (*p* < 0.05) decreases MCP‐1, ET‐1, and VEGF expression compared to non‐treated OGD group. Full unedited blot for Figure 3D is provided as Figure S2.

### 3.4 In T1DM stroke rats, 3 µg/kg VT treatment significantly decreases inflammatory factor expression and apoptosis in the IBZ

**Figure 4:**
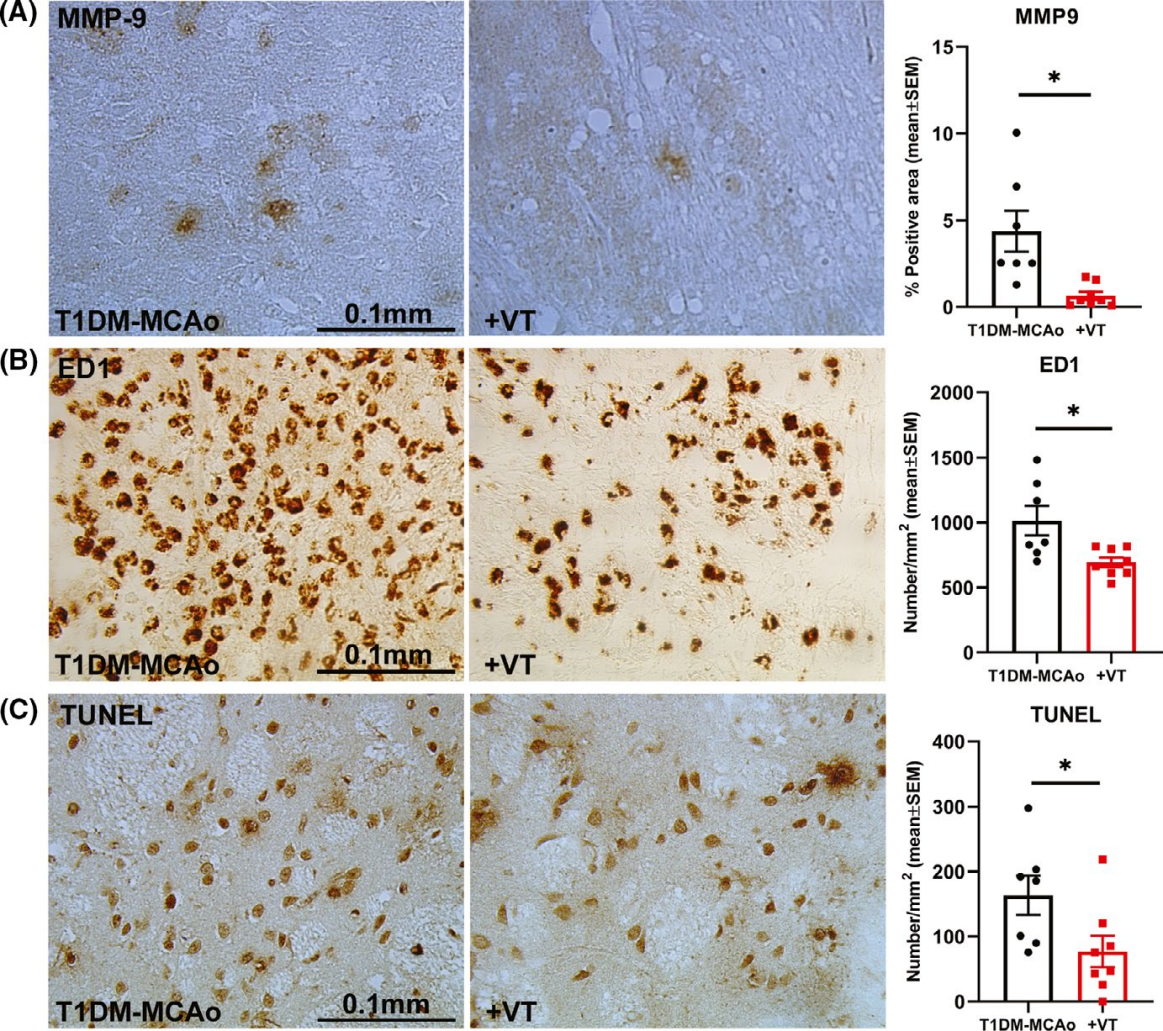
Immunostaining with matrix metalloproteinase 9 (MMP9), M1 macrophage marker ED1, and apoptosis marker TUNEL was employed to evaluate inflammation and cell death in the ischemic brain, and data were analyzed using unpaired Student's *t*‐test with Welch's correction. Treatment of T1DM stroke with 3 µg/kg VT initiated 24 h after stroke (A) significantly decreases inflammatory factor MMP9 expression, (B) significantly decreases the number of ED1 positive M1 macrophages, and (C) significantly decreases the number of TUNEL‐positive apoptotic cells in the ischemic border zone after stroke in T1DM rats compared to PBS‐treated T1DM‐MCAo group. *N* = 7–8 animals per group. *p < 0.05

MMP9 is a neuroinflammatory factor known to regulate white matter injury and pathological cerebrovascular remodeling in diabetic stroke.^38^, ^39^ Inflammatory cells such as macrophages are a major source for MMP9 under pathological conditions.^40^ Therefore, to evaluate the effects of VT treatment on inflammatory responses after stroke, we measured the expression of MMP9 in IBZ and M1 macrophage number using ED1 immunostaining. We also evaluated TUNEL expression to determine the effect of VT treatment on apoptosis. Our data show that 3 µg/kg VT treatment significantly decreases inflammatory factor MMP9 expression (Figure 4A, *p* < 0.05), reduces the number of macrophages (ED1, Figure 4B, *p *< 0.05), and decreases the number of apoptotic cells (TUNEL, Figure 4C, *p *< 0.05) in the IBZ after stroke in T1DM rats compared to PBS‐treated control group.

### 3.5 VT significantly decreases inflammatory factor expression in vitro

**Figure 5:**
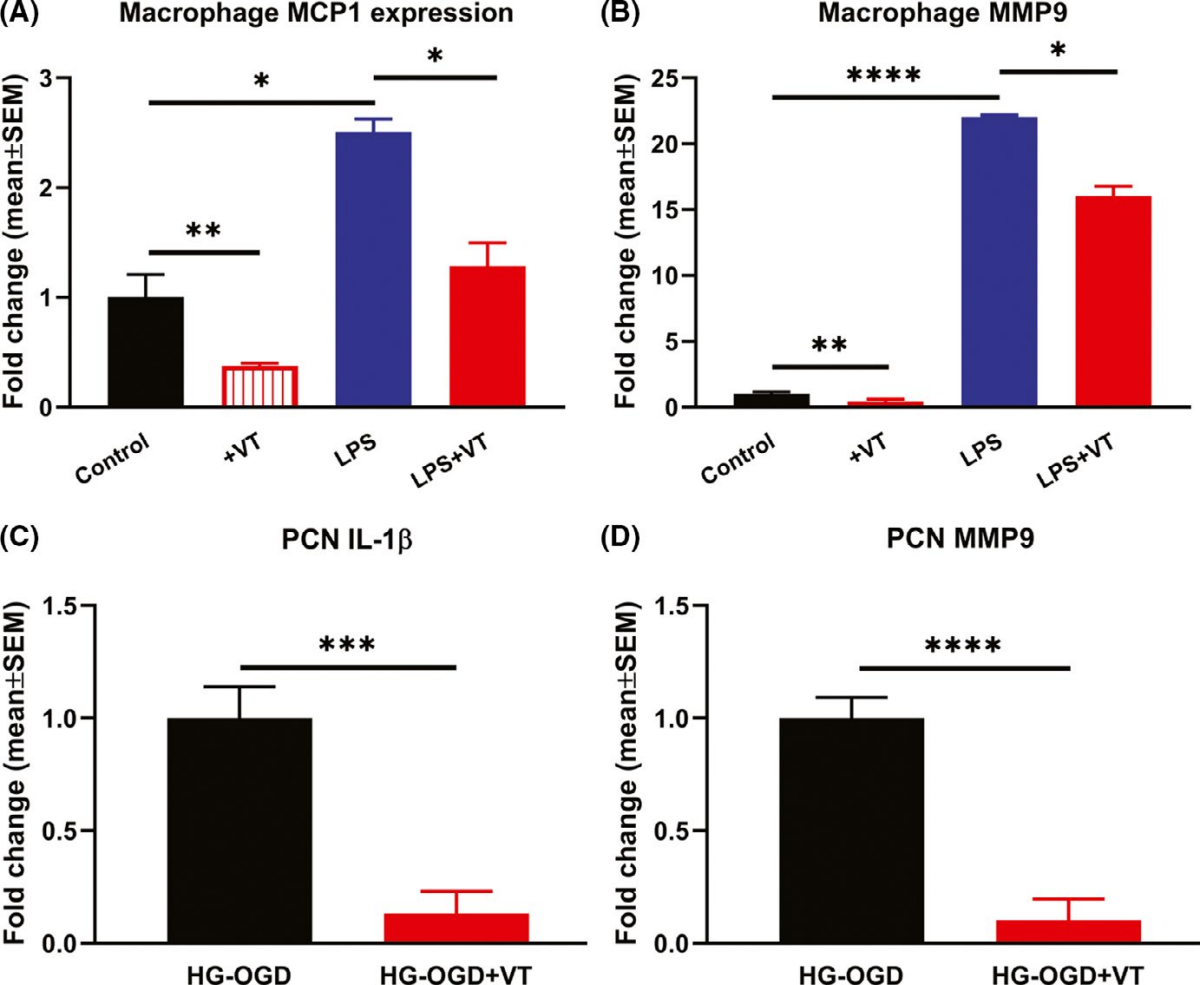
Primary bone marrow derived macrophages were subjected to LPS stimulation to induce M1 polarized macrophage and test whether VT treatment decreases inflammatory factor secretion using RT‐PCR. Macrophages subjected to LPS activation and treated with 10 nM VT exhibit significantly reduced expression of (A) MCP‐1 and (B) MMP9 compared to untreated cells. (C‐D) To test the effect of VT treatment on neuroinflammation, primary cortical neurons (PCNs) were subject to conditions of high glucose (HG) and oxygen glucose deprivation and RT‐PCR was used to evaluate IL‐1β and MMP9 expression. PCNs subjected to conditions of HG and oxygen glucose deprivation and treated with 10 ng/ml VT exhibit significantly decreased expression of inflammatory factors IL‐1β and MMP9 compared to non‐treated control. *N* = 3/group. *p < 0.05, **p < 0.01, ***p < 0.001, ****p < 0.0001

LPS stimulation is known to polarize macrophages to M1 phenotype and secrete inflammatory mediators and cytotoxic factors, while exerting minimal effect on anti‐inflammatory (M2) phenotype.^41^, ^42^ Therefore, to test whether VT treatment decreases secretion of inflammatory factors by M1 polarized macrophages, we employed LPS stimulation model to induce polarized macrophage and inflammatory factor secretion. Figure 5A,B shows that macrophages treated with VT have significantly (*p* < 0.05) reduced expression of MCP‐1 and MMP9 compared to untreated cells. To test the effects of VT treatment on neuronal inflammatory responses, we used PCR to assess inflammatory factor expression of PCNs subjected to OGD and HG conditions. Figure 5C,D shows that under conditions of HG and ischemic stress, PCNs treated with 10 ng/ml VT exhibit significantly (*p* < 0.001) decreased expression of inflammatory factors IL‐1β and MMP9 compared to non‐treated control.

## 4 DISCUSSION

In the current study, we show that 3 µg/kg VT treatment of stroke in T1DM rats significantly improves neurological function and decreases ischemic lesion volume and apoptosis which may be mediated in part by enhanced white matter and vascular remodeling and reduced inflammatory responses. We have previously reported the neuroprotective effects of pre‐stroke VT treatment in T1DM stroke.^13^ The results of the present study provide evidence for therapeutic benefits of delayed 24 h onset of VT treatment for stroke in rats with T1DM.

Ischemic lesion volume is an important predictor of functional outcome after stroke with larger lesions corresponding to worse stroke outcome.^43^, ^44^ Diabetes and stroke are both known to trigger neuronal apoptosis, and dysregulated apoptosis in the IBZ can contribute to increased cell death and larger infarct area following stroke.^44^, ^45^, ^46^ Ang1 has previously been shown to reduce infarct volume in mice subjected to focal cerebral embolic ischemia.^7^ A meta‐analysis study of 11 pre‐clinical stroke studies reported that Ang1 upregulation significantly decreases lesion volume and improves BBB integrity following transient ischemic stroke in rodents.^14^ Treatment with Ang1 has also been shown to reduce neuronal cell death by inhibiting the upregulation of pro‐apoptotic factors in vitro.^47^ In our previous study, we showed that pre‐treatment of stroke with VT reduced lesion volume and apoptosis which translated to improved functional outcome.^13^ In the present study, we show that delayed and long‐term VT treatment of T1DM stroke significantly reduces lesion volume and apoptosis compared to control T1DM‐stroke rats. The reduction in lesion volume may contribute to improved neurological function in VT‐treated rats compared to control T1DM stroke rats. The neuroprotective effects of Ang1 treatment in non‐DM rodents have been previously demonstrated.^7^, ^8^, ^14^, ^48^, ^49^, ^50^ In non‐DM rats subject to 2 h of transient MCAo stroke, intravascular administration of recombinant Ang1 (1 μg/kg) once every 12 h starting immediately after reperfusion significantly decreases neurological severity score, decreases infarct volume, attenuates BBB leakage, and increases the expression of tight junction proteins in the ischemic brain.^49^, ^50^ Similarly, in non‐DM mice subject to an embolic MCAo model, treatment with recombinant Ang1 protein or recombinant adenoviruses expressing Ang1 prior to stroke significantly decreased BBB leakage and reduced ischemic lesion volume.^7^ Our data also indicate that 3 µg/kg VT treatment in non‐DM stroke significantly (*p* < 0.05) improves neurological function and decreases ischemic lesion volume compared to control non‐DM stroke rats. Future studies employing neuroimaging techniques such as MRI^51^ to evaluate stroke outcome are warranted.

Ischemic stroke is an established cause of white matter injury.^35^, ^36^, ^52^ Increased white matter injury is associated with poor long‐term functional outcomes after stroke in experimental models of DM,^31^, ^39^ as well as after traumatic brain injury.^33^, ^53^ Diabetes reduces the proliferation and survival of oligodendrocyte progenitor cells and impairs the generation of oligodendrocytes in white matter lesions after stroke.^31^, ^54^ Therapeutics, that promote the differentiation and maturation of oligodendrocyte progenitor cells and attenuate microglia/macrophage activation and neuroinflammation, improve long‐term neurological outcome following stroke,^35^ as well as hypoxia‐ischemia injury.^34^ Ang1 promotes angiogenesis which can ultimately improve white matter integrity through increased blood flow and oxygen supply to ischemic tissue.^55^ Treatment with Ang1 has been shown to prevent white matter damage after spinal cord injury.^56^ In addition, Ang1 has been shown to exert neurorestorative effects by causing an increase in neuronal structural protein expression in a model of T2DM‐induced peripheral neuropathy.^57^ Our previous study has shown that an increase in Ang1 may be partially responsible for the increase of white matter remodeling in T1DM stroke animals treated with human umbilical cord blood cells.^58^ Therefore, increasing Ang1 likely promotes both vascular and white matter remodeling in diabetic stroke animals. Our data indicate that VT treatment of T1DM stroke significantly increases axon and myelin density, improves white matter integrity, and increases oligodendrocyte progenitor cells numbers compared to control T1DM stroke rats which may contribute to improved stroke outcome.

Chronic exposure to HG impairs neurovascular coupling, BBB integrity, and cerebrovascular patterning.^59^ Diabetic stroke animals exhibit severe vascular injury, arteriosclerosis, and worse functional outcomes compared to wild‐type stroke animals.^10^, ^60^ Angiopoietin's exert distinct roles from VEGF in diabetes and in post‐stroke angiogenesis.^15^, ^61^ Compared to non‐diabetic mice, in the chronic phase after ischemic injury, T1DM mice exhibit significantly increased proangiogenic factors VEGFa and Ang2 expression, while the expression level of maturation and stabilization factors Ang1, PDGF‐β, and TGF‐β was not significantly altered.^11^ In diabetic animals, dramatically increased VEGF signaling is linked to the generation of immature and unstable vessels with increased vascular dysfunction and vascular leakage.^11^, ^37^, ^61^ VEGF inhibition at delayed time point after stroke not only attenuates BBB permeability but also improves functional outcome in diabetic animals.^62^ In addition to promoting endothelial cell survival and vessel stabilization, Ang1 also attenuates VEGF‐induced transendothelial permeability and plasma leakage.^61^, ^63^, ^64^ In diabetic wound healing, adenoviral‐mediated overexpression of Ang‐1 aided wound closure via increased neovascularization, increased capillary density and decreased inflammatory cells, independent of VEGF signaling.^65^ In T1DM rats, neovascularization in subcutaneous inserted Matrigel with pre‐added VEGF indicated pathological angiogenesis with incomplete/abnormal neovascular endothelial structure, while treatment with intravenous Ang‐1 adenovirus increased functional vascular tube like structures with intact endothelial and tight junction structure, and reduced inflammatory cell infiltration.^66^ In our previous study, we show that Ang1 contributes to HUCBC‐induced improvement in vascular remodeling in T1DM stroke animals.^58^ Our data indicate that VT treatment significantly decreases endothelial cell VEGF expression under HG and ischemic conditions as well as improves capillary tube formation under HG conditions when compared to non‐treated control cells. In vivo, our data indicate that treatment of stroke in T1DM animals increases vascular density as indicated by the increased number of vessels in the IBZ compared to control T1DM stroke rats. Therefore, VT treatment induced increase in angiogenesis and vascularization may also contribute to improvement in neurological function in T1DM stroke rats.

ET‐1 is a potent vasoconstrictor peptide that is produced by endothelial cells and contributes to vascular injury through inhibitory effects on nitric oxide production.^67^, ^68^ ET‐1 is elevated in the circulation of diabetic patients and implicated in vascular dysfunction.^68^, ^69^ In diabetic mice, endothelial cell ET‐1 overexpression exacerbates hyperglycemia induced endothelial dysfunction by increasing vascular oxidative stress.^67^ ET‐1 can directly stimulate and amplify inflammatory factor induced MCP‐1 production in endothelial cells.^70^ Ang1 is vasoprotective, inhibits endothelial apoptosis, and attenuates vascular inflammation.^71^ Ang1 dose dependently reduced the release of ET‐1 from cultured endothelial cells.^71^ Our data indicate that under conditions of HG and OGD, VT treatment decreases endothelial cell death and decreases ET‐1 and MCP‐1 expression compared to control group. Therefore, VT treatment decreases significantly decreases vascular inflammation.

Neuroinflammation is an important factor in the progression of diabetic stroke pathology which exacerbates white matter and vascular damage and contributes to neurological function deficits.^72^, ^73^ There are numerous pro‐ and anti‐inflammatory cytokines which are significantly increased in the first 24 h after ischemic stroke and inhibiting macrophage/microglia from acquiring a pro‐inflammatory phenotype and limiting the subsequent cytokine storm favors long‐term neurological recovery in mice.^74^, ^75^ While subsets of activated microglia promote remyelination by facilitating the generation, migration, and maturation of oligodendrocyte progenitor cells and oligodendrocytes, prolonged activation and microglia derived TNF‐α and IL‐1β can be cytotoxic to normal brain cells and exacerbate demyelination in the injured brain.^75^ Chronic and progressive inflammation indicated by elevated hippocampal IL‐1β and TNF‐α expression is known to play a role in diabetes‐related dementia.^76^ The expression of various pro‐inflammatory cytokines is increased in patients with diabetes, whereas anti‐inflammatory cytokines are downregulated.^77^, ^78^ MMP9 plays important roles in inflammation and contributes to increased BBB permeability after stroke.^79^ Particularly in diabetic stroke, MMP9 contributes to aggravated white matter injury and worse stroke outcome.^39^ MMP9 and other plasma inflammatory factors are modified in the acute stages of stroke inflammation, which suggests their involvement in the development and progression of stroke pathology.^80^ Ang1 treatment has been previously shown to counteract inflammatory responses and protect endothelial cell integrity.^58^ In our previous study, we showed that VT treatment of T1DM stroke decreases brain expression of inflammatory factors such as MCP‐1, RAGE, and TNF‐α compared to control T1DM stroke rats.^13^ Our data indicate that T1DM stroke rats treated with VT exhibit significantly reduced MMP9 expression and decreased numbers of M1 macrophages in the ischemic brain compared to control T1DM stroke rats. We also found that VT treatment significantly reduces the expression of MCP‐1 and MMP9 in macrophages subjected to LPS activation when compared to untreated cells in vitro. VT treatment also reduces MMP9 and IL‐1β in PCNs subjected to hypoxia under HG conditions. Therefore, reduced inflammation in the ischemic brain likely contributes to VT treatment induced improved stroke outcome in T1DM stroke rats.

## 5 CONCLUSIONS

Delayed daily treatment with 3 µg/kg of VT for 14 days after stroke improves neurological function and reduces infarct volumes in T1DM animals. Delayed VT treatment promotes white matter integrity and vascular remodeling and reduces the expression of inflammatory factors which may contribute to improved stroke outcome in T1DM rats. These data provide evidence for investigating the therapeutic efficacy of VT for stroke treatment in the diabetic population.

## CONFLICT OF INTEREST

None.

## AUTHOR CONTRIBUTIONS

Formal analysis and investigation: Poornima Venkat, Alex Zacharek, Julie Landschoot‐Ward, Lauren Culmone, Ruizhuo Ning, Linlin Liang; Manuscript preparation: Poornima Venkat, Lauren Culmone; Supervision: Michael Chopp.

## Supporting information

Fig S1‐2

## Data Availability

All relevant data is contained within the manuscript.
