# Supplementary material for: Treatment with an Angiopoietin‐1 mimetic peptide promotes neurological recovery after stroke in diabetic rats
Source: CNS Neurosci Ther. 2020 Dec 21;27(1):48–59. doi: 10.1111/cns.13541 (PMC7804913; doi:10.1111/cns.13541)

**Supplementary figure 1:** (A) Neurological function was evaluated on days 1, 7 and 14 after stroke using modified neurological severity score (mNSS) test and analyzed using one-way ANOVA- Bonferroni's multiple comparisons test. Treatment of WT- stroke rats with 3µg/kg VT initiated 24h after stroke and administered once daily (i.p) significantly improves neurological functional outcome compared to control MCAo rats. (B) At 14 days after stroke, lesion volume was evaluated using H&E staining and analyzed using unpaired Student’s t-test with Welch’s correction. Treatment of stroke with 3µg/kg VT initiated 24h after stroke significantly decreases lesion volume compared to control MCAo rats. N=5-6 animals per group. *p<0.05, **p<0.01.


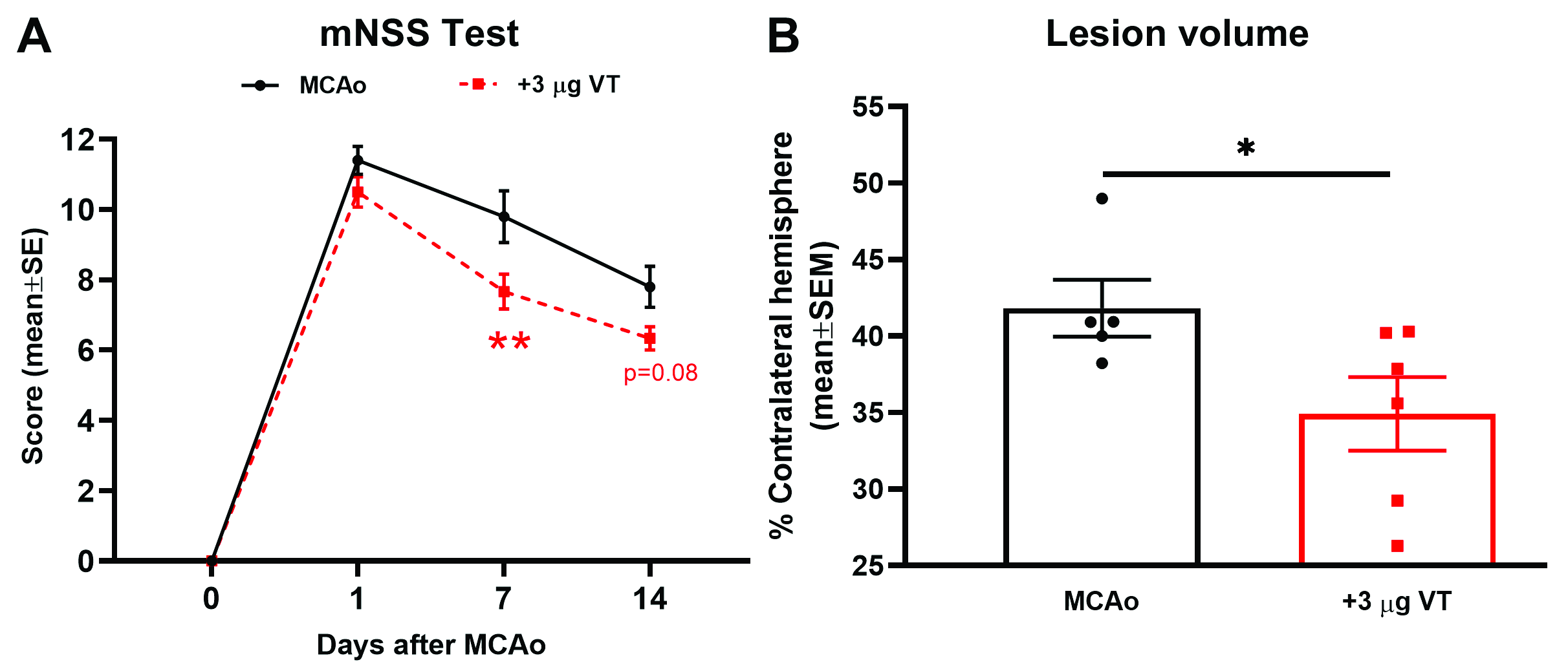


**Supplementary figure 2:** Full unedited blot for Figure 3D. To evaluate the differential expression of angiogenic factors by endothelial cells under conditions of high glucose and oxygen glucose deprivation with and without VT treatment, an angiogenesis protein array was performed.


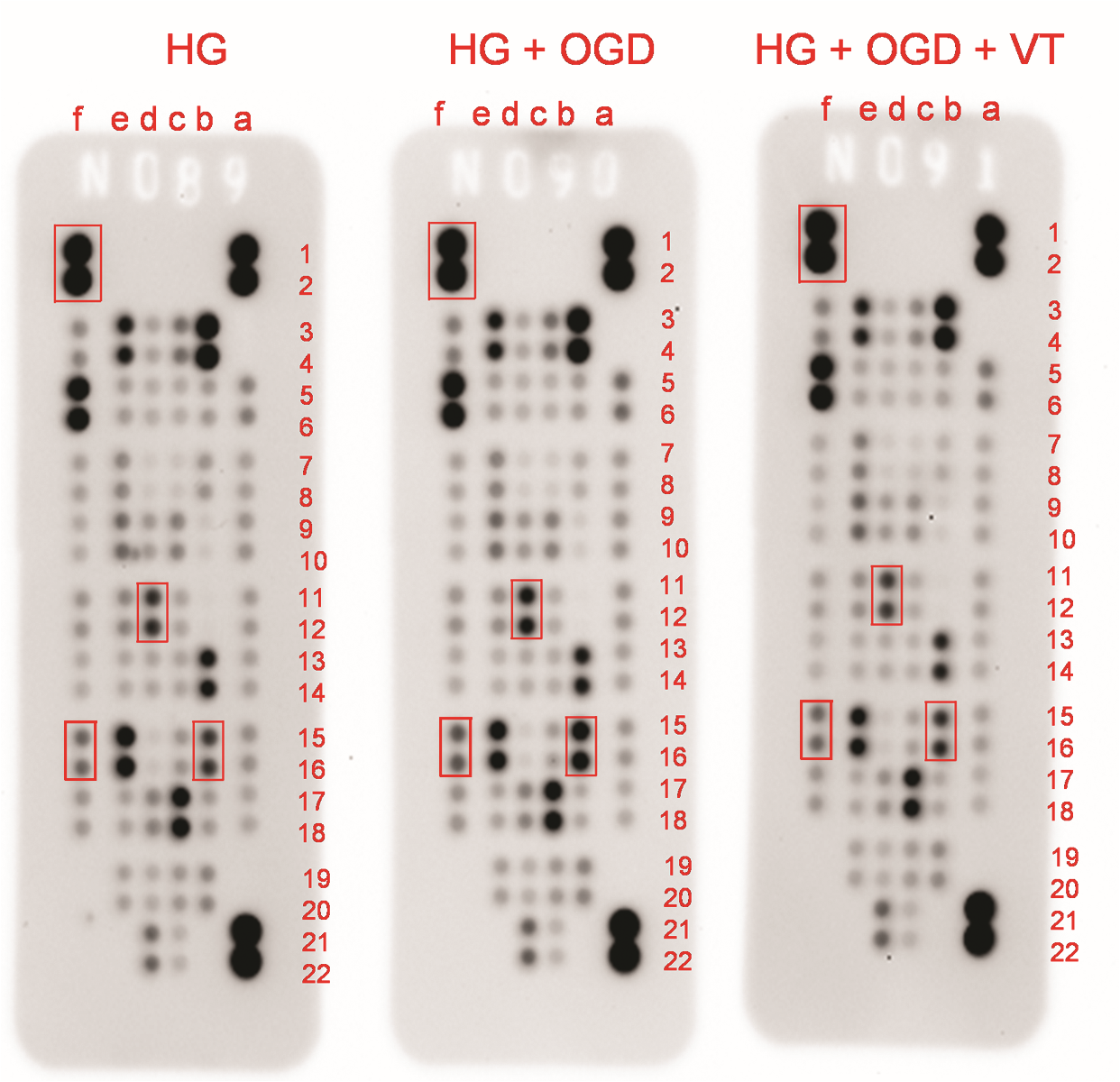

Supplement: Supplementary file 1 — Fig S1‐2 [file CNS-27-48-s001.docx]
